# A prospective randomized self-controlled study of LASIK combined with accelerated cross-linking for high myopia in Chinese: 24-month follow-up

**DOI:** 10.1186/s12886-022-02491-y

**Published:** 2022-06-24

**Authors:** Ruilan Dong, Yu Zhang, Yifei Yuan, Yan Liu, Yuexin Wang, Yueguo Chen

**Affiliations:** 1 Department of Ophthalmology, Peking University Third Hospital, Beijing, China; 2 Beijing Key Laboratory of Restoration of Damaged Ocular Nerve, Peking University Third Hospital, Beijing, China

**Keywords:** LASIK Xtra, LASIK, High myopia, Regression

## Abstract

**Background:**

To assess the visual and refractive outcomes of femtosecond laser-assisted in situ keratomileusis (FS-LASIK) concurrent with accelerated cross-linking (LASIK Xtra) compared with conventional FS-LASIK (convLASIK) for high myopia in Chinese individuals.

**Methods:**

In this prospective, randomized, fellow-eye comparison study, 25 patients with high myopia were treated randomly with LASIK Xtra in one eye and convLASIK in the other. A 24-month follow-up was conducted, and the main outcome measures included uncorrected distance visual acuity (UDVA), corrected distance visual acuity (CDVA), manifest refraction spherical equivalent (MRSE) and corneal tomography.

**Results:**

The UDVA was 0.09 ± 0.15 logMAR in the LASIK Xtra group, which was significantly worse than that in the convLASIK group 1 day postoperatively (*P* = .001), but the difference became nonsignificant from 1 week after surgery. The efficacy index was 0.88 ± 0.18 in the LASIK Xtra eyes and 0.99 ± 0.13 in the convLASIK eyes at 24 months (*P* = .028). Throughout the follow-up period, a slight myopic shift in the MRSE and keratometry values were observed in both groups without significant intergroup differences. The coefficient of determination was 0.9982 in the LASIK Xtra eyes and 0.9987 in the convLASIK eyes. The corneal density was significantly higher, and demarcation lines were visible in the first 6 months in LASIK Xtra eyes, but both signs of cross-linking gradually disappeared during follow-up. No severe complications were detected in either group.

**Conclusions:**

LASIK Xtra showed comparable safety and predictability with convLASIK for high myopia in Chinese, but lower efficacy and no greater stability was observed up to 24-month follow-up.

## Introduction

Femtosecond laser-assisted in situ keratomileusis (FS-LASIK) is a common and affordable refractive surgery for a large number of people, especially those with aesthetic or occupational demands. However, the circumferential corneal flap has been proven to weaken the integrity and rigidity of the cornea as much as 14% to 33% [1, 2], which may result in post-LASIK ectasia with a prevalence of 0.02% to 0.6% [3–5]. By increasing the combinations of covalent bonds among collagen fibres, corneal collagen cross-linking (CXL) acts as a therapeutic method for keratoconus and post-LASIK ectasia [6, 7]. To prevent ectasia from the very beginning of the procedure, accelerated CXL was introduced as a prophylactic method into the conventional FS-LASIK (convLASIK) procedure [8].

Many studies have focused on demonstrating the greater stability achieved with CXL concurrent with LASIK (LASIK Xtra) than with convLASIK, but no consensus has been reached. Some studies have suggested that LASIK Xtra shows reduced myopic drift and better keratometric stability than convLASIK [8–11], while others have reported no significantly different refractive or keratometric outcomes between the two procedures [12–17]. In addition, few studies have conducted self-controlled research in populations with high myopia for more than 12 months. To further evaluate the necessity of adjuvant CXL in the myopic LASIK procedure, we performed LASIK Xtra and convLASIK randomly in high myopic fellow eyes and followed up for 24 months in the present study.

## Patients and methods

### Patients

This prospective self-controlled randomized cohort study enrolled 50 eyes of 25 patients with high myopia and/or myopic astigmatism in the Peking University Third Hospital (PUTH) from May 2019 to January 2020. This study adhered to the tenets of the Declaration of Helsinki and was approved by the Institutional Review Board of the PUTH.

The inclusion criteria were as follows: 1) age > 18 years; 2) preoperative spherical refractive error from -6.00 D to -14.00 D; 3) stable refractive error for at least 1 year; 4) cessation of wearing soft contact lenses for 1 week or rigid contact lenses for 3 weeks; 5) no ocular disease except for ametropia; and 6) no history of ocular surgery or trauma. The exclusion criteria included 1) surgical contraindications such as explicit keratoconus or forme fruste keratoconus; 2) anisometropia of more than 1.00 D; 3) predicted residual stromal bed thickness of less than 280 μm; 4) active ocular inflammation; 5) systemic diseases or medication affecting wound healing; and 6) pregnancy or lactation.

All patients signed informed consent forms before the surgery after receiving an explanation of the nature and consequences of the study and underwent convLASIK in one eye (selected randomly) and LASIK Xtra in the fellow eye. The randomization was performed by sealed opaque envelopes.

### Preoperative examinations

**Fig. 1:**
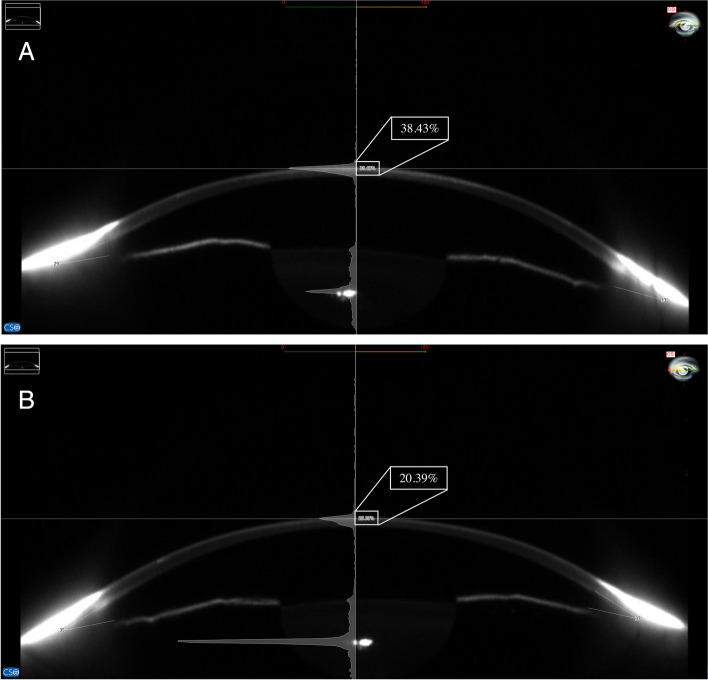
Measurement of corneal density. **A** The eye that underwent LASIK Xtra in one patient. **B** The fellow eye in the same patient, which underwent convLASIK

Detailed ocular examinations prior to the surgical procedures included measurements of uncorrected distance visual acuity (UDVA), corrected distance visual acuity (CDVA), manifest refraction, cycloplegic refraction and intraocular pressure, slit-lamp examination, dilated funduscope examination, corneal topography (Vario Topolyzer; Alcon Laboratories Inc, Fort Worth, TX, USA), corneal tomography (Sirius; CSO, Florence, Italy), endothelial cell count (Nidek; Nidek Co, Tokyo, Japan) and partial optical coherence interferometry (IOL master; Zeiss, Jena, Germany). The topographic parameters from the Topolyzer device were used for iris registration and automatic kappa angle and cyclotorsion compensation during operation, while those from the Sirius device were adopted for follow-up and statistical analysis. The corneal density was measured manually in triplicate by an experienced technician using a Scheimpflug image from the Sirius device, and the average value was expressed as a percentage of the maximum value (Fig. 1). The same optician for each examination was masked regarding which eye was treated with LASIK Xtra.

### Surgical procedures

All surgeries were performed by two experienced ophthalmologists (YZ and YC) under topical anaesthesia. Flaps were created by a WaveLight FS200 femtosecond laser (Alcon Laboratories Inc, Fort Worth, TX, USA) with a thickness of 110 μm and diameter of 8.5 to 9.0 mm. The hinge position was superior. Ablation was performed by a WaveLight EX500 excimer laser (Alcon Laboratories Inc, Fort Worth, TX, USA) with an optical zone of 6.0 to 6.5 mm, and the nomogram supplied by the manufacturer was adopted with a refractive target of plano-correction for all eyes. For the LASIK Xtra group, the bare stromal bed was saturated with 0.22% riboflavin diluted with saline (Vibex Xtra; Avedro Inc, Waltham, Massachusetts, USA) for 90 s. The flap was carefully protected so as not to contact the Vibex Xtra. The riboflavin was rinsed thoroughly with balanced saline solution afterwards, and the flap was repositioned properly. Irradiation was then accomplished using a KXL system (Avedro Inc, Waltham, Massachusetts, USA) with a UVA fluence of 30 mW/cm^2^ for 90 s (total energy 2.7 J/cm^2^).

### Postoperative care and follow-up

Postoperatively, topical medications were administered as follows in both groups: 0.1% fluorometholone (FML; Allergan Pharmaceuticals, Dublin, Ireland) four times daily and tapering for 1 month, 0.5% levofloxacin (Cravit; Santen Pharmaceutical (China) Co Ltd, Jiangsu, China) four times daily for 2 weeks and artificial tears four times daily for 1 month.

All patients were asked to test their visual acuity one day and one week after surgery. Further follow-ups were performed at 1, 3, 6, 12 and 24 months. Follow-up examinations included measurements of UDVA and CDVA, manifest refraction, slit-lamp examination, corneal tomography and endothelial cell count (ECC).

### Statistical analysis

Visual acuity values measured by a Snellen chart were converted to logarithm of the minimum angle of resolution (logMAR) accordingly. Efficacy and safety indices were calculated as the ratio of postoperative UDVA and CDVA, respectively, over preoperative CDVA. Data were analysed using SPSS (Version 24; IBM Corporation, Armonk, NY, USA). Normality of continuous variables was tested using the Shapiro–Wilk test. Normally distributed data are presented as the mean ± standard deviation, and nonnormally distributed data are presented as the median. Normally distributed variables were compared using the paired t test, while the Wilcoxon signed-rank test was used in the absence of a normal distribution. Categorical variables are presented as frequencies and percentages and were compared using the chi-squared test or Fisher’s exact test. Intragroup difference analysis during the follow-up period was performed using repeated ANOVA or the Friedman test. The Pearson method was used for correlation analysis. A *P* value of < 0.05 was considered statistically significant.

## Results

The mean age was 27.7 ± 7.0 (range 18 to 42) years, and 22 (88%) were females among the 25 patients. There was no significant difference in baseline characteristics between the two groups (Table 1). Due to the COVID-19 pandemic, the follow-up rate was not satisfactory. Twenty-one patients were followed up for 1 month, and 18 patients were followed up at 3 and 24 months. At 6 months and 12 months after surgery, 13 and 11 patients were followed up, respectively. Therefore, given the statistical difficulty caused by the unbalanced data, we carried the data of 12 months for the 5 patients who failed to follow up at 6 months forward to fill in the missing values. The data of the remaining 6 patients at 12 months were excluded. The average follow-up time of the newly combined group was 7.7 months, but we still recorded this time point as the 6th month for convenience of comparison with similar studies.

**Table 1:** Baseline characteristics

Parameter	LASIK Xtra (*n* = 25)	convLASIK(*n* = 25)	*P*
UDVA (logMAR)	1.22 ± 0.23 (0.82, 1.70)	1.21 ± 0.23 (0.82, 1.70)	.372
CDVA (logMAR)	-0.04 ± 0.05 (-0.18, 0.06)	-0.05 ± 0.04 (-0.08, 0.02)	.409
MRSE (D)	-8.76 ± 1.52 (-13.75, -7.00)	-8.64 ± 1.50 (-13.25, -6.50)	.219
Cylinder (D)	-1.17 ± 0.85 (-3.50, 0.00)	-1.14 ± 0.75 (-3.00, 0.00)	1.000
Flat K (D)	43.00 ± 1.57 (39.41, 46.38)	43.13 ± 1.45 (39.63, 46.13)	.378
Steep K (D)	44.39 ± 1.67 (41.06, 48.31)	44.49 ± 1.52 (41.10, 47.74)	.656
Mean K (D)	43.68 ± 1.55 (40.22, 47.33)	43.80 ± 1.41 (40.35, 46.92)	.478
Thinnest corneal thickness (μm)	549 ± 28 (499, 593)	548 ± 26 (507, 591)	.384
ECC (/mm^2^)	3032.3 ± 277.8 (2551.6, 3739.3)	3009.3 ± 212.0 (2573.2, 3437.1)	.903

*LASIK Xtra* Femtosecond laser-assisted in situ keratomileusis combined with intraoperative cross-linking, *convLASIK* Conventional femtosecond laser-assisted in situ keratomileusis, *UDVA* Uncorrected distance visual acuity, *CDVA* Corrected distance visual acuity, *MRSE* Manifest refraction spherical equivalent, *ECC* Endothelial cell count, *logMAR* Logarithm of the minimum angle of resolution, *D* Diopters

### Safety

There was no postsurgical ectasia or other severe intra- or postoperative complications. Mild haze was observed in 16 (76%) eyes of the LASIK Xtra group at the 1-month follow-up and remained in 12 eyes at 3 months. The haze was barely resolved until 6 months after surgery.

**Fig. 2:**
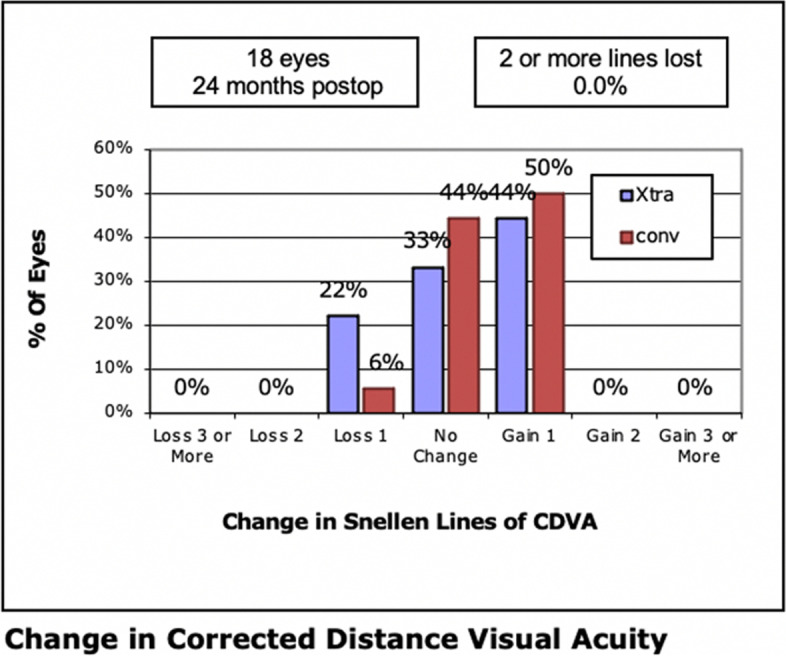
Change in Snellen lines of CDVA at 24 months in the LASIK Xtra and convLASIK groups

At the 24-month follow-up, 8 (44%) eyes of the LASIK Xtra group versus 9 (50%) eyes of the convLASIK group gained 1 Snellen line of CDVA, while 4 (22%) eyes and 1 (6%) eye lost 1 line in the LASIK Xtra and convLASIK groups, respectively (*P* = 0.367 and 0.338, respectively) (Fig. 2).

The safety index remained close to 1 throughout the 24 months in both groups, and there was no statistically significant difference between the two groups at each follow-up (Table 2).

**Table 2:** Postoperative visual and refractive outcomes

**Parameter**	**Month 1** **(*n* = 21)**	**Month 3** **(*n* = 18)**	**Month 6** **(*n* = 18)**	**Month 24** ***(n* = 18)**	***P***
**UDVA (logMAR)**					
Xtra	-0.02 ± 0.05	-0.01 ± 0.09	-0.02 ± 0.05	0.00 ± 0.11	.755
Conv	-0.02 ± 0.06	-0.02 ± 0.05	-0.02 ± 0.04	-0.05 ± 0.04	.338
P	.706	1.000	.900	.119	
**CDVA (logMAR)**					
Xtra	-0.06 ± 0.05	-0.11 ± 0.05	-0.05 ± 0.05	-0.04 ± 0.04	.096
Conv	-0.04 ± 0.05	-0.05 ± 0.05	-0.04 ± 0.06	-0.08 ± 0.04	.353
P	.220	.474	.673	.202	
**MRSE (D)**					
Xtra	0.11 ± 0.32	0.01 ± 0.39	-0.01 ± 0.31	-0.16 ± 0.33	.392
Conv	0.20 ± 0.49	0.11 ± 0.31	0.10 ± 0.45	0.11 ± 0.29	.765
*P*	.097	.114	.540	.455	
**Cylinder (D)**					
Xtra	-0.18 ± 0.20	-0.25 ± 0.22	-0.30 ± 0.22	-0.32 ± 0.34	.507
Conv	-0.32 ± 0.25	-0.23 ± 0.26	-0.20 ± 0.33	-0.32 ± 0.30	.166
*P*	.746	.942	.720	.557	
**Efficacy index**					
Xtra	0.97 ± 0.14	0.92 ± 0.18	0.94 ± 0.17	0.88 ± 0.18	.094
Conv	0.97 ± 0.17	0.97 ± 0.11	0.99 ± 0.10	0.99 ± 0.13	.948
*P*	.730	.194	.795	.028*	
**Safety index**					
Xtra	1.03 ± 0.16	1.08 ± 0.17	0.99 ± 0.16	0.99 ± 0.16	.099
Conv	0.99 ± 0.17	1.04 ± 0.14	1.01 ± 0.13	1.08 ± 0.13	.171
*P*	.498	.619	.395	.112	
**Thinnest corneal thickness (μm)**					
Xtra	385 ± 24	389 ± 23	398 ± 21	416 ± 13	< .001**
Conv	399 ± 25	406 ± 27	404 ± 22	412 ± 19	.002**
*P*	.001**	.001**	.023*	.793	
**ECC (/mm**^**2**^**)**					
Xtra	2806.7 ± 184.9	2935.5 ± 238.5	2887.4 ± 109.1	3211.3 ± 270.1	.098
Conv	2765.3 ± 234.8	2860.0 ± 62.0	2812.1 ± 108.8	3143.3 ± 161.6	.058
*P*	.979	.553	.448	.271	

*Xtra* Femtosecond laser-assisted in situ keratomileusis combined with intraoperative cross-linking, *Conv* Conventional femtosecond laser-assisted in situ keratomileusis, *UDVA* Uncorrected distance visual acuity, *CDVA* Corrected distance visual acuity, *MRSE* manifest refraction spherical equivalent, *ECC* Endothelial cell count, *logMAR* Logarithm of the minimum angle of resolution, *D* Diopters. *P < .05, **P < .01

### Efficacy

One day after surgery, a better UDVA (-0.02 ± 0.07 logMAR) was achieved in convLASIK eyes than in LASIK Xtra eyes (0.09 ± 0.15 logMAR, *P* = 0.001). However, the difference became nonsignificant at 7 days postoperatively (*P* = 0.084) and all subsequent follow-ups between the two groups. At 24 months, the logMAR UDVA was 0.00 ± 0.11 in the LASIK Xtra group and -0.05 ± 0.04 in the convLASIK group (*P* = 0.119) (Table 2).

**Fig. 3:**
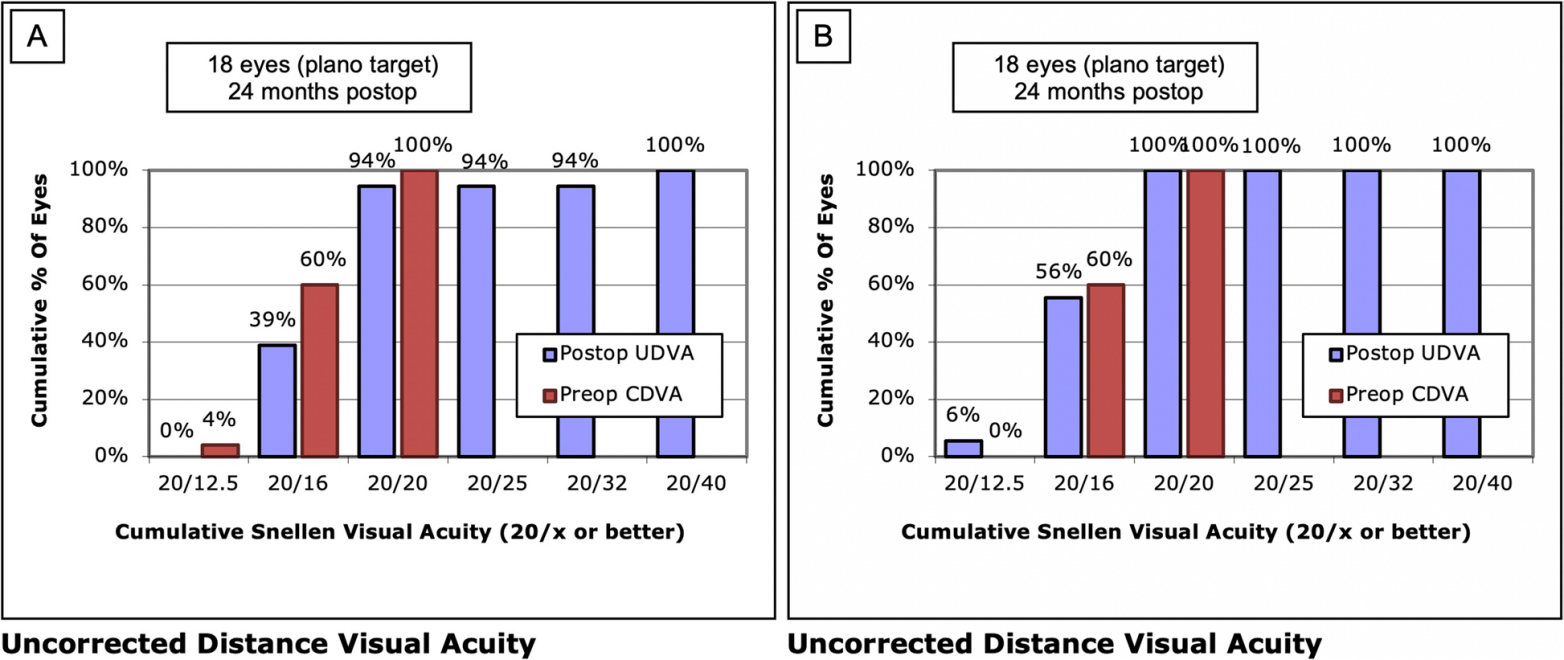
Cumulative Snellen visual acuity preoperatively and at 24 months in the LASIK Xtra (**A**) and convLASIK (**B**) groups

A UDVA of 20/20 or better was achieved for 20 (95.23%), 16 (88.89%), 17 (94.44%) and 17 (94.44%) eyes in the LASIK Xtra group at 1, 3, 6 and 24 months respectively after surgery, while in the convLASIK group, 18 (85.71%), 16 (88.89%), 17 (94.44%) and 18 (100.00%) eyes, respectively, achieved a 20/20 or better UDVA (*P* = 0.606, 1.000, 1.000 and 1.000, respectively) (Fig. 3).

There was no significant difference in the efficacy index between the two groups except for the last follow-up. At 24 months, the efficacy index was 0.88 ± 0.18 in LASIK Xtra eyes, which was significantly lower than 0.99 ± 0.13 in convLASIK eyes (*P* = 0.028) (Table 2).

### Stability

**Fig. 4:**
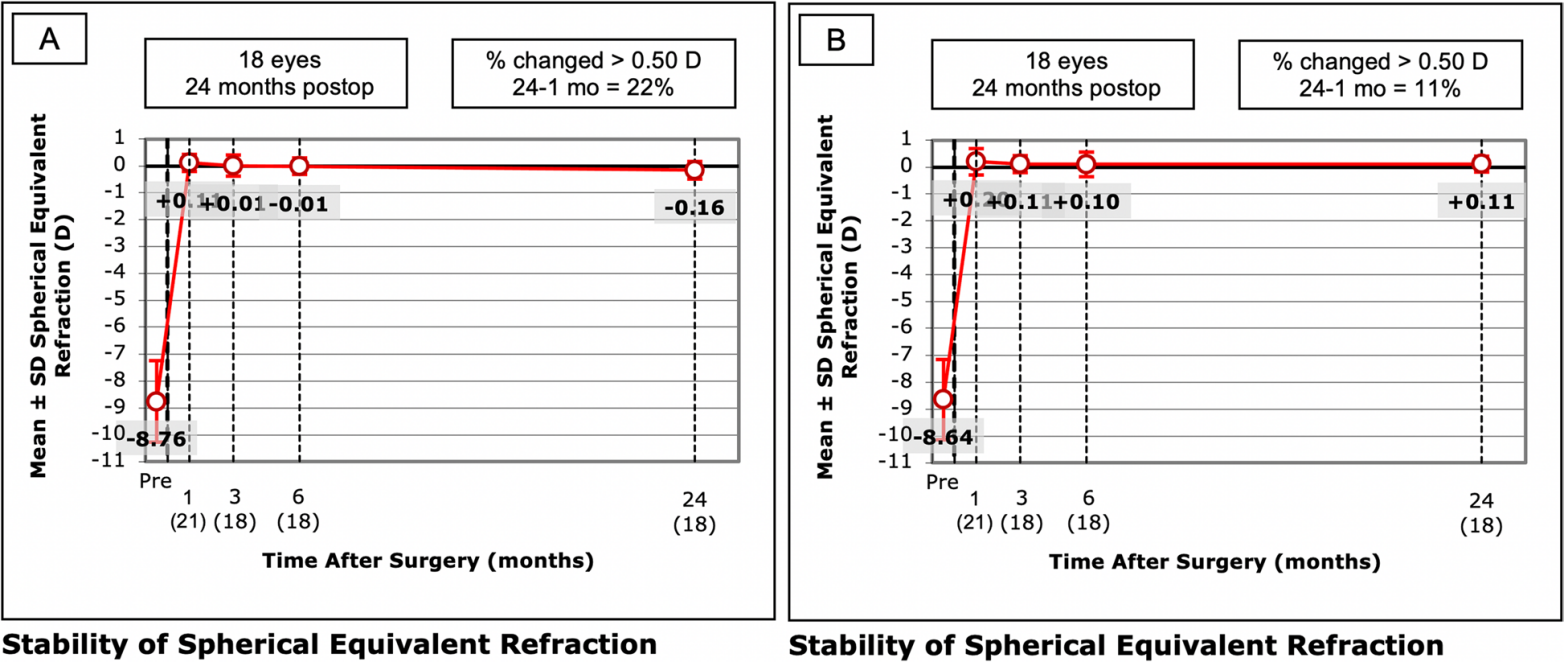
Stability of spherical equivalent refraction in the LASIK Xtra (**A**) and convLASIK (**B**) groups

The MRSE was measured at each follow-up point, and the results are listed in Table 2. There was no statistically significant difference between the two groups preoperatively and postoperatively (Fig. 4). Both groups exhibited a slight trend of regression over time. MRSE drifted from 0.11 ± 0.32 D at one month after surgery to -0.16 ± 0.33 D at 24 months after surgery in the LASIK Xtra group and from 0.20 ± 0.49 D to 0.11 ± 0.29 D in the convLASIK group, but no statistically significant difference in refractive shift from 1 month to 24 months was observed between the two groups (-0.18 ± 0.41 D in LASIK Xtra vs. -0.14 ± 0.33 D in convLASIK, *P* = 0.774).

In the postoperative follow-up, flat K (*P* = 0.034), steep K (*P* = 0.015) and mean K (*P* = 0.020) in the LASIK Xtra group and flat K (*P* = 0.041) in the convLASIK group increased significantly. However, the K values did not vary significantly between the two groups throughout the follow-up period, nor did the changes in K values from 1 month or 3 months to 24 months postoperatively (Table 3).

**Table 3:** Postoperative keratometric outcomes

Parameter	LASIK Xtra	convLASIK	Difference in mean between groups	Difference in change between groups		
				Month 3	Month 6	Month 24
**Flat K (D)**						
Month 1	35.59 ± 2.03	35.54 ± 1.50	.308	.599	.565	.290
Month 3	35.79 ± 1.93	35.77 ± 1.49	.894	-	.524	.769
Month 6	35.74 ± 1.99	35.70 ± 1.59	.828	-	-	.640
Month 24	36.02 ± 1.84	35.88 ± 1.53	.938	-	-	-
**Steep K (D)**						
Month 1	36.09 ± 1.98	35.91 ± 1.47	.465	.268	.307	.670
Month 3	36.36 ± 1.76	36.12 ± 1.53	.313	-	.903	.815
Month 6	36.30 ± 1.77	36.09 ± 1.74	.218	-	-	.634
Month 24	36.60 ± 1.75	36.18 ± 1.56	.286	-	-	-
**Mean K (D)**						
Month 1	35.84 ± 2.00	35.73 ± 1.48	.416	.410	.394	.570
Month 3	36.07 ± 1.85	35.94 ± 1.51	.654	-	.735	.799
Month 6	36.01 ± 1.88	35.90 ± 1.66	.447	-	-	.598
Month 24	36.31 ± 1.79	36.03 ± 1.55	.541	-	-	-

*LASIK Xtra* Femtosecond laser-assisted in situ keratomileusis combined with intraoperative cross-linking, *convLASIK* Conventional femtosecond laser-assisted in situ keratomileusis, *D* Diopters

### Predictability

**Fig. 5:**
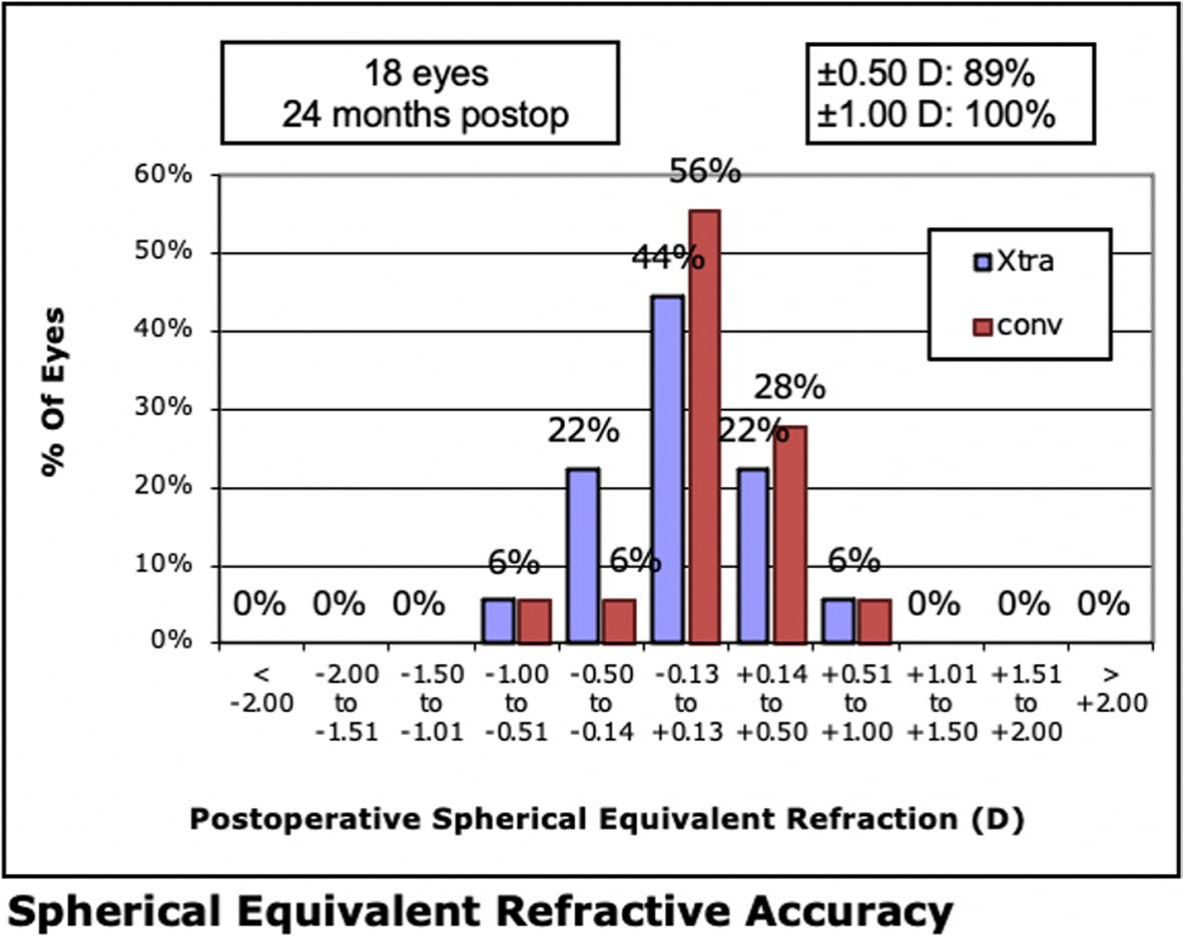
Spherical equivalent refraction at 24 months in the LASIK Xtra and convLASIK groups

At 1, 3, 6, and 24 months, 19 (90.48%), 16 (88.89%), 16 (88.89%) and 16 (88.89%) eyes, respectively, achieved an MRSE within ± 0.50 D of the attempted MRSE in the LASIK Xtra group, while in convLASIK eyes, 18 (85.71%), 17 (94.44%), 15 (83.33%) and 16 (88.89%) eyes achieved an MRSE within ± 0.50 D, respectively (Fig. 5). The proportions were not significantly different.

**Fig. 6:**
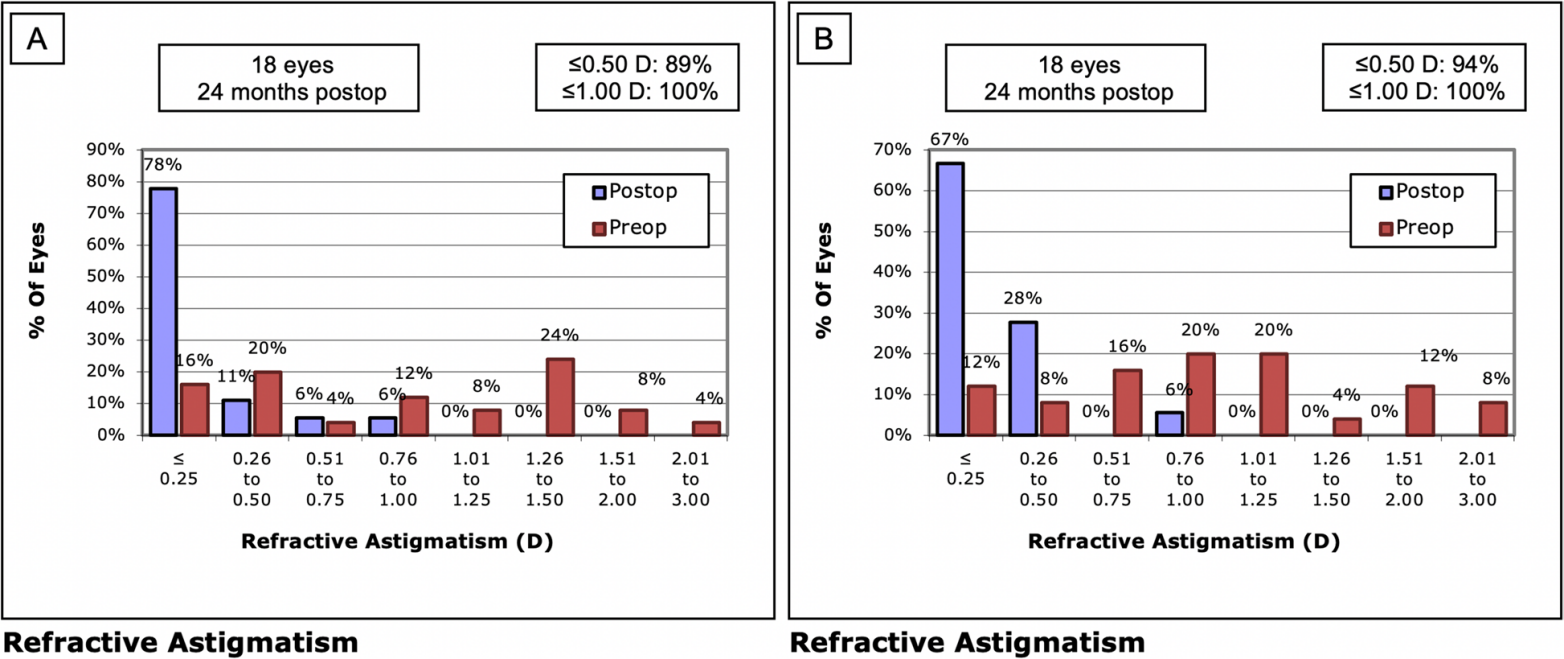
Refractive astigmatism preoperatively and at 24 months in the LASIK Xtra (**A**) and convLASIK (**B**) groups

Refractive astigmatism was reduced significantly from -1.17 ± 0.85 D preoperatively to -0.32 ± 0.34 D postoperatively in LASIK Xtra eyes (*P* = 0.001) and from -1.14 ± 0.75 D to -0.32 ± 0.30 D in convLASIK eyes (*P* < 0.001) (Table 2), but there was no significant difference between the two groups throughout the follow-up period. The accuracy of cylinder correction was comparable between the two groups, with 78% of eyes in the LASIK Xtra group and 67% in the conventional group achieving a correction within ± 0.25 D at the 24-month follow-up (*P* = 0.711) (Fig. 6).

**Fig. 7:**
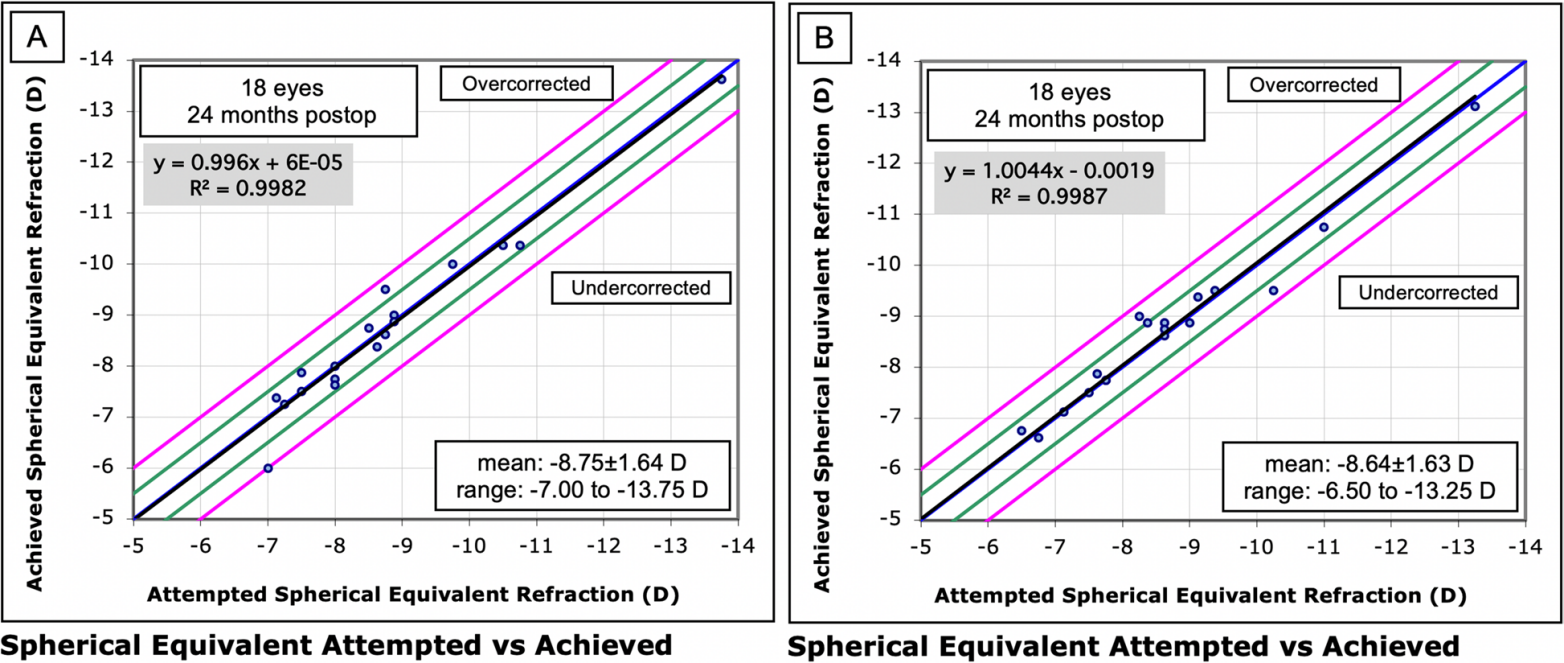
Spherical equivalent attempted vs. achieved in the LASIK Xtra (**A**) and convLASIK (**B**) groups

Both groups showed a strong correlation between the attempted and achieved spherical equivalent refraction, as shown in scatter plots (Fig. 7). The coefficient of determination between the attempted and achieved spherical equivalent refraction was 0.9982 in LASIK Xtra eyes and 0.9987 in convLASIK eyes.

### Corneal thickness and endothelial cell count

**Fig. 8:**
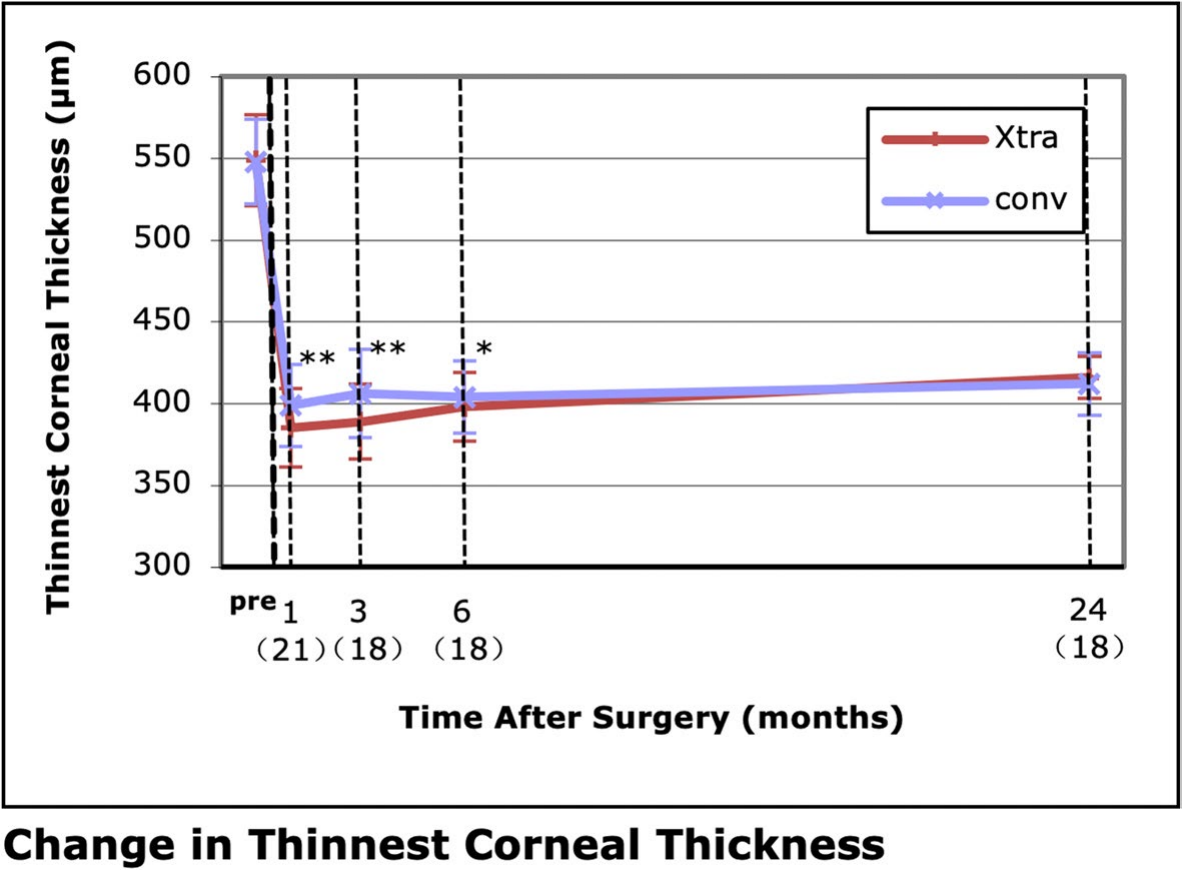
Change in thinnest corneal thickness. *P < .05; **P < .01

The thinnest corneal thickness was 385 ± 24 μm, 389 ± 23 μm and 389 ± 23 μm at 1, 3 and 6 months, respectively, in the LASIK Xtra group, which was accordingly lower than the 399 ± 25 μm, 406 ± 27 μm and 404 ± 22 μm in the convLASIK group (*P* = 0.001, 0.001 and 0.023, respectively) (Fig. 8). Similar levels were achieved between the two groups at 24 months after the operation (416 ± 13 μm in LASIK Xtra vs. 412 ± 19 μm in convLASIK, *P* = 0.793).

There was no statistically significant intergroup difference in ECC at any observation point (Table 2).

### Corneal density

**Fig. 9:**
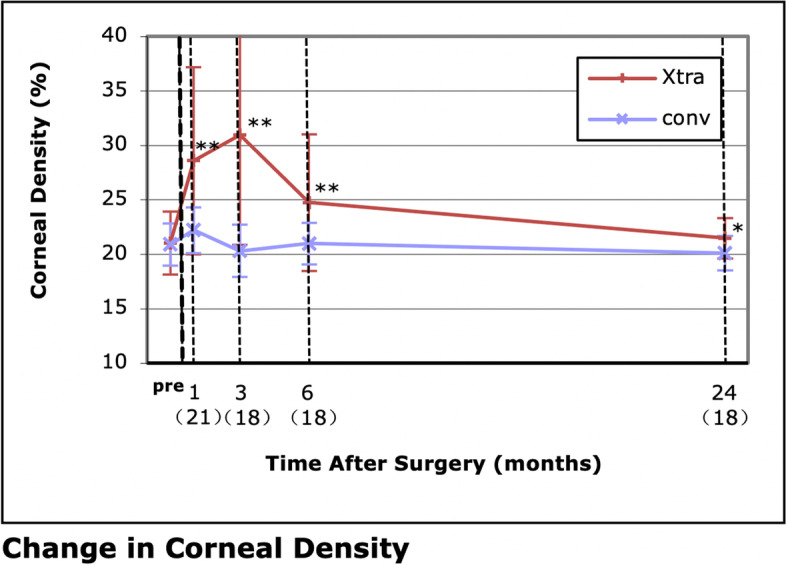
Change in corneal density. *P < .05; **P < .01

Demarcation lines were visible at 1 month after surgery in 61.5% LASIK Xtra eyes, most clearly defined at 3 months and disappeared at approximately 6 months. The density of the cornea, measured by the Sirius device and exhibited as a percentage, was significantly higher in the LASIK Xtra group at 1 (*P* = 0.001), 3 (*P* < 0.001), 6 (*P* = 0.002) and even 24 (*P* = 0.043) months, but the difference between the two groups gradually narrowed (Fig. 9).

## Discussion

By enhancing corneal rigidity, CXL has been proven effective in slowing down or even arresting progression in keratoconus or postsurgical ectasia. Thus, LASIK Xtra, which involves simultaneous accelerated CXL and LASIK, is assumed to be a prophylactic procedure for populations at relatively high dilated risks, including young age, a thin predicted residual stromal bed and a history of heredity [4, 18]. Some studies have shown favourable outcomes regarding the safety, efficacy, predictability and stability of LASIK Xtra versus convLASIK [8–11], while in the present study, comparable but not significantly better refractive outcomes were observed in the LASIK Xtra group.

The biological effect is proportionate to the total irradiation energy, as assumed by the Bunsen-Roscoe Law of Reciprocity [19, 20]; however, the irradiation protocol ranges from 1.4 to 5.4 J/cm^2^ in previous studies, and no consensus has been achieved [21]. An excessive dose may result in continued corneal flattening, which would be favourable in treating keratoconus but not in prophylaxis for postsurgical ectasia [22]. We adopted 2.7 J/cm^2^, which is one of the most common designs, with the goal of balancing between reinforcing the cornea and reducing side effects such as corneal oedema and opacity. The dose adopted in this study was effective because demarcation lines were visible and the corneal density measured by tomography was significantly higher in the LASIK Xtra group throughout the follow-up period.

LASIK Xtra is a safe procedure for highly myopic populations. No severe intra- or postoperative complications have been observed in any studies, although some have reported mild haze [8, 13, 15, 16, 23, 24], diffuse lamellar keratitis [13, 16, 24] and epithelial erosions [16], which disappeared from 1 day to 12 months after surgery. The intra- or intergroup ECC was not significantly different throughout the follow-up period [9, 10, 13, 14, 17, 24]. In line with previous studies, we found no severe adverse events in the LASIK Xtra group. The safety index at 24 months after surgery was comparable between the two groups, and the ECC remained stable during follow-up.

In regard to efficacy, the LASIK Xtra group may perform worse shortly after surgery. In this study, the LASIK Xtra protocol had inferior refractive outcomes one day after surgery, which did not recover until one week postoperatively. Tomita [17] and colleagues also reported a better UDVA in the convLASIK group at -0.11 ± 0.11 logMAR, compared with the -0.06 ± 0.08 logMAR in the LASIK Xtra group 1 day postoperatively (*P* = 0.008). A similar conclusion was drawn by Chan (*P* = 0.001) [15], and in other studies the inferiority in LASIK Xtra eyes lasted until 1 month after surgery [12, 14, 16]. The transient delayed visual rehabilitation in the LASIK Xtra group may be attributed to corneal oedema or haze induced by irradiation at an early stage, which was proven by the fact that equal or even better UDVA was achieved over longer follow-up when the oedema or haze has eased [12, 14–16]. While in contrast to previous studies which generally reported nonsignificantly lower efficacy index in the LASIK Xtra group [14, 17], we observed significantly lower efficacy index in LASIK Xtra eyes at 24 months. We supposed that the worse performance following LASIK Xtra may be due to the fact that prophylactic accelerated CXL only increases the tissue reaction but failes to sufficiently strengthen the cornea as therapeutic CXL.

In general, LASIK with and without CXL both achieved good predictability with a strong correlation between the attempted and achieved MRSE. Low J.R [13]. reported that 72% of eyes achieved an MRSE within ± 0.50 D in the LASIK Xtra group and 84% in the convLASIK group at the 3-month follow-up. Chan [15] and associates achieved similar outcomes 6 months after surgery. In studies with longer follow-up periods, the percentage of eyes that achieved an MRSE within ± 0.50 D seemed to be slightly higher in the LASIK Xtra group, although no significant difference was observed [9, 10, 12, 17]. The present study arrived at the same conclusion, which indicated that the effect of adjunctive CXL is predictable and that there is no need to adjust the nomogram.

Stability is the core focus in the evaluation of LASIK Xtra compared to convLASIK; however, previous studies failed to report evidence sufficient enough to support LASIK Xtra. In the present study, the stability concerning both MRSE and K values in the LASIK Xtra group showed no advantage over that in the convLASIK group throughout the 24-month follow-up. In researches by Tomita [17] and Tan [11], nonsignificant difference was also reported in the MRSE and keratometry between the two groups. Kohnen and associates [12] observed a more conspicuous trend towards myopic regression in the conventional procedure (*P* = 0.09), but which did not differ significantly from LASIK Xtra (*P* = 0.86). Kanellopoulos made similar conclusions of the nonsignificant difference concerning myopic regression between the two procedures through both 12-month (*P* = 0.063) [10] and 24-month (*P* = 0.065) [9] follow-ups, but indicated significantly increased keratometric stability in LASIK Xtra eyes (*P* = 0.032 [9] and 0.039 [10], respectively). This difference may be related to the fact that the average MRSE of the populations included in these two studies is relatively lower than that of other studies, so that less corneal tissue was ablated. Considering that the postoperative ectasia could happen in several years, the nonsignificant effect of Xtra procedure may attribute to relatively short period under observation. However, it also suggests that the total irradiation energy of existing protocol may not be sufficient to make a difference. It is worth further exploring new protocols with greater total energy or better permeability of riboflavin to maximize the effectiveness of CXL in refractive surgery, while the balance between complications and stability needs to be considered.

The innovation of the flap creation technique using a femtosecond laser rather than a microkeratome has improved the predictability of flap thickness and reduced the risk of severe flap complications [25, 26]. In addition, the surgical population is now strictly screened so that only patients with a residual stromal bed above 280 μm are eligible for LASIK. We interpreted the comparable refractive outcomes between the additional and standard protocols as evidence supporting the reliability and safety of FS-LASIK alone even for highly myopic populations. As for highly myopic patients with borderline topography abnormalities, phakic intraocular lens implantation might be a better choice providing better visual outcomes and safety [27, 28].

The limitations of our study included the relatively high rate of loss to follow-up due to the implementation of COVID-19 pandemic prevention and control guidelines; also, there is a lack of consecutive biomechanical examinations and accurate densitometry provided by Oculus Pentacam, especially at the early postoperative stage, when they are more likely to differ. Moreover, the 24-month follow-up is greater than the average level of previous studies, but a longer follow-up is still needed, considering that post-LASIK ectasia may occur from 1 week to several years postoperatively [29, 30].

In conclusion, we conducted a randomized fellow-eye controlled study in a higher myopic population with a longer follow-up period than previous studies. The present study confirmed LASIK Xtra to be as safe as convLASIK, and favourable predictability was also achieved. However, no superior stability was observed in LASIK Xtra eyes over convLASIK eyes, which was the initial motivation for combining CXL with the standard LASIK procedure. In addition, simultaneous CXL may weaken the advantages of LASIK, such as rapid visual rehabilitation, and increase relift difficulty for enhancement. We therefore tend to regard CXL as a treatment for post-LASIK ectasia rather than a prophylaxis unless more compelling evidence with long-term follow-up can be published.

## Data Availability

The datasets generated and/or analysed during the current study are not publicly available due to limitations of ethical approval involving the patient data and anonymity but are available from the corresponding author on reasonable request.

## Abbreviations

FS-LASIK: Femtosecond laser-assisted in situ keratomileusis
LASIK Xtra: Femtosecond laser-assisted in situ keratomileusis concurrent with accelerated cross-linking
convLASIK: Conventional FS-LASIK
CXL: Corneal collagen cross-linking
UDVA: Uncorrected distance visual acuity
CDVA: Corrected distance visual acuity
MRSE: Manifest refraction spherical equivalent
ECC: Endothelial cell count
logMAR: Logarithm of the minimum angle of resolution
PUTH: Peking University Third Hospital
